# Brain amyloid load, subjective memory complaints, and cognitive trajectories in older individuals at risk for dementia

**DOI:** 10.1111/ene.16436

**Published:** 2024-08-12

**Authors:** Gazi Saadmaan, Anette Hall, Tiia Ngandu, Nina Kemppainen, Francesca Mangialasche, Gayle M. Wittenberg, Anna Matton, Juha O. Rinne, Miia Kivipelto, Alina Solomon

**Affiliations:** ^1^ Department of Neurology, Institute of Clinical Medicine University of Eastern Finland Kuopio Finland; ^2^ Division of Clinical Geriatrics, Center for Alzheimer Research, Department of Neurobiology, Care Sciences, and Society Karolinska Institute Stockholm Sweden; ^3^ Population Health Unit Finnish Institute for Health and Welfare Helsinki Finland; ^4^ Turku PET Center University of Turku Turku Finland; ^5^ Division of Clinical Neurosciences Turku University Hospital Turku Finland; ^6^ Medical Unit Aging, Theme Inflammation, and Aging Karolinska University Hospital Stockholm Sweden; ^7^ Neuroscience, Data Science, & Digital Health Janssen Research & Development Titusville New Jersey USA; ^8^ Institute of Public Health and Clinical Nutrition University of Eastern Finland Kuopio Finland; ^9^ Ageing Epidemiology Research Unit, School of Public Health Imperial College London London UK

**Keywords:** Alzheimer disease, amyloid, clinical trial, cognition, PiB‐PET

## Abstract

**Background and Purpose:**

This study evaluated associations of brain amyloid with 2‐year objective and subjective cognitive measures in a trial‐ready older general population at risk for dementia.

**Methods:**

Forty‐eight participants in the Finnish Geriatric Intervention Study to Prevent Cognitive Impairment and Disability underwent ^11^C‐Pittsburgh compound B (PiB) positron emission tomography (PET) scans and assessment of cognition (modified Neuropsychological Test Battery [NTB]) and subjective memory complaints (Prospective and Retrospective Memory Questionnaire).

**Results:**

Mean age was 71.4 ± 5.06 years, and 20 participants (42%) had positive baseline PiB‐PET scans. Amyloid positivity was associated with lower NTB executive function at baseline and less favorable 2‐year NTB total score and memory trajectories, but not with other objective or subjective cognitive measures. Overall, there was little cognitive decline during 2 years.

**Conclusions:**

Amyloid accumulation may affect objective but not necessarily subjective cognition from a very early at‐risk stage, although substantial decline likely requires >2 years to occur.

## INTRODUCTION

Amyloid‐beta (Aβ) accumulation, a key neuropathological change in Alzheimer disease (AD), can occur decades before dementia onset [1]. However, the significance of Aβ accumulation for cognitive decline is still debated [2]. Aβ‐targeted disease‐modifying therapies are assumed to be more effective if started early [2], but the Aβ–cognition relation in older cognitively normal individuals is not fully clear, complicating recruitment of suitable clinical trial participants. Associations between amyloid status and cognition among cognitively normal older adults have been investigated in mostly cross‐sectional but also in longitudinal studies, with varying results. One meta‐analysis reported Aβ‐related cognitive impairment in global cognition, visuospatial function, processing speed, episodic memory, and executive function, and also Aβ‐related decline over time in global cognition, semantic memory, visuospatial function, and episodic memory [1]. A recent systematic review found inconsistent results across studies investigating associations between Aβ‐positivity and various cognitive, measures with some studies reporting significant associations, whereas others reported no associations [3]. The International Working Group (IWG) 2021 recommendations for AD diagnosis have also emphasized that Aβ‐positive cognitively normal individuals may not necessarily experience cognitive decline [2].

Subjective cognitive complaints (SCC) may represent the first AD symptoms [4]. Although there are reports of associations of Aβ accumulation with SCC [5, 6], a meta‐analysis of the prevalence of Aβ pathology in people without dementia reported that the frequency of AD biomarker profiles was similar between people with and without SCC [7]. Longitudinally, one study linked Aβ pathology to increasing complaints over time [8], whereas another study showed no association [9] in older cognitively normal individuals.

In this study, we investigated cross‐sectional and longitudinal associations of Aβ accumulation on ^11^C‐Pittsburgh compound B (PiB) positron emission tomography (PET) imaging with subjective and objective cognitive performance in older adults at risk for dementia, but without substantial impairment. Study participants were part of the Finnish Geriatric Intervention Study to Prevent Cognitive Impairment and Disability (FINGER). We hypothesized that Aβ accumulation would be related to both subjective and objective cognitive performance across multiple domains. These associations have not been previously investigated in trial‐ready general at‐risk populations. Analyzing a “trial‐ready” population is particularly important for addressing challenges of finding the right individuals at the right time and for the right interventions.

## METHODS

### Study population

The FINGER trial protocol and primary results have been published [10, 11]. Briefly, FINGER was a 2‐year randomized controlled trial of a multidomain lifestyle intervention (nutrition advice, exercise program, cognitive training and social activities, and vascular/metabolic risk monitoring) compared to regular health advice among 1260 older individuals from the general Finnish population. Eligibility criteria included age 60–77 years, cardiovascular risk factors, aging and dementia (CAIDE) Dementia Risk Score ≥6 points, and cognitive performance around the mean level or slightly lower than expected for age. Individuals with dementia or substantial impairment were excluded. The primary outcome was change in cognition assessed with a modified version of the Neuropsychological Test Battery (NTB) [12, 13].

The FINGER (ClinicalTrials.gov identifier: NCT01041989) study was approved by the coordinating ethics committee of the Hospital District of Helsinki and Uusimaa. Written informed consent was obtained from participants during screening and baseline visits. Separate written informed consent was obtained for the PET scans.

### PiB‐PET assessment

Recruitment and protocol of the FINGER PiB‐PET substudy (Turku PET Center) were previously described (Data S1) [14]. This substudy population (*N* = 48) was not significantly different from the rest of the FINGER trial, except for somewhat older mean age (70.8 vs. 69.3 years) [14]. Brain scans were conducted in connection with the FINGER baseline visit.

Participants underwent 3‐T brain magnetic resonance imaging, and a dynamic scan was performed from 60 to 90 min (3 × 10‐min frames) after ^11^C‐PiB‐PET injection (Philips Ingenuity TF PET/MR, Amsterdam, the Netherlands). Two experienced readers assessed the scans visually as Aβ± based on regional patterns of PiB retention and following consensus agreement. PiB‐positive individuals had ^11^C‐PiB‐PET retention in at least one cortical region characteristically affected in AD, whereas PiB negative individuals had only nonspecific ^11^C‐PiB‐PET retention in white matter.

### Cognitive assessment

NTB was administered at baseline and 1‐ and 2‐year visits by trained study psychologists blinded to randomization group allocation. Prespecified primary (NTB total score) and secondary cognitive outcomes (NTB memory, executive function, and processing speed scores) were calculated as composite *z*‐scores standardized to the baseline mean and SD, with higher scores showing better performance, as previously described [14]. The Prospective and Retrospective Memory Questionnaire was used for self‐assessment of SCC at the 6‐month and 2‐year trial visits; a total score and prospective and retrospective memory scores were calculated, with higher scores indicating more subjective complaints [14].

### Statistical analyses

Baseline characteristics of Aβ± groups were compared using *t*‐test or χ^2^ test as appropriate. Associations between Aβ± status at baseline and NTB measures were assessed with mixed effects regression models with maximum likelihood estimation. In the main models, change in cognitive scores was analyzed as a function of Aβ± status at baseline, Aβ status × time interaction, randomization group, time, and group × time interaction. In these models, the Aβ status term showed the Aβ–cognition association at baseline, whereas the Aβ status × time interaction term showed the association between baseline Aβ and longitudinal cognitive change. Further adjustments of the main models were conducted separately for age, sex, or education. Results are reported as estimates and 95% confidence interval (CIs).

Associations between Aβ± status at baseline and SCC were analyzed using linear regression models adjusted for age, sex, and education with further adjustment for randomization group for longitudinal analyses of change in SCC. SCC change was calculated as the difference between 24‐ and 6‐month values, and zero‐skewness log‐transformed.

Statistical analyses were conducted with Stata 15 statistical software; *p* < 0.05 was considered statistically significant.

## RESULTS

**FIGURE 1:**
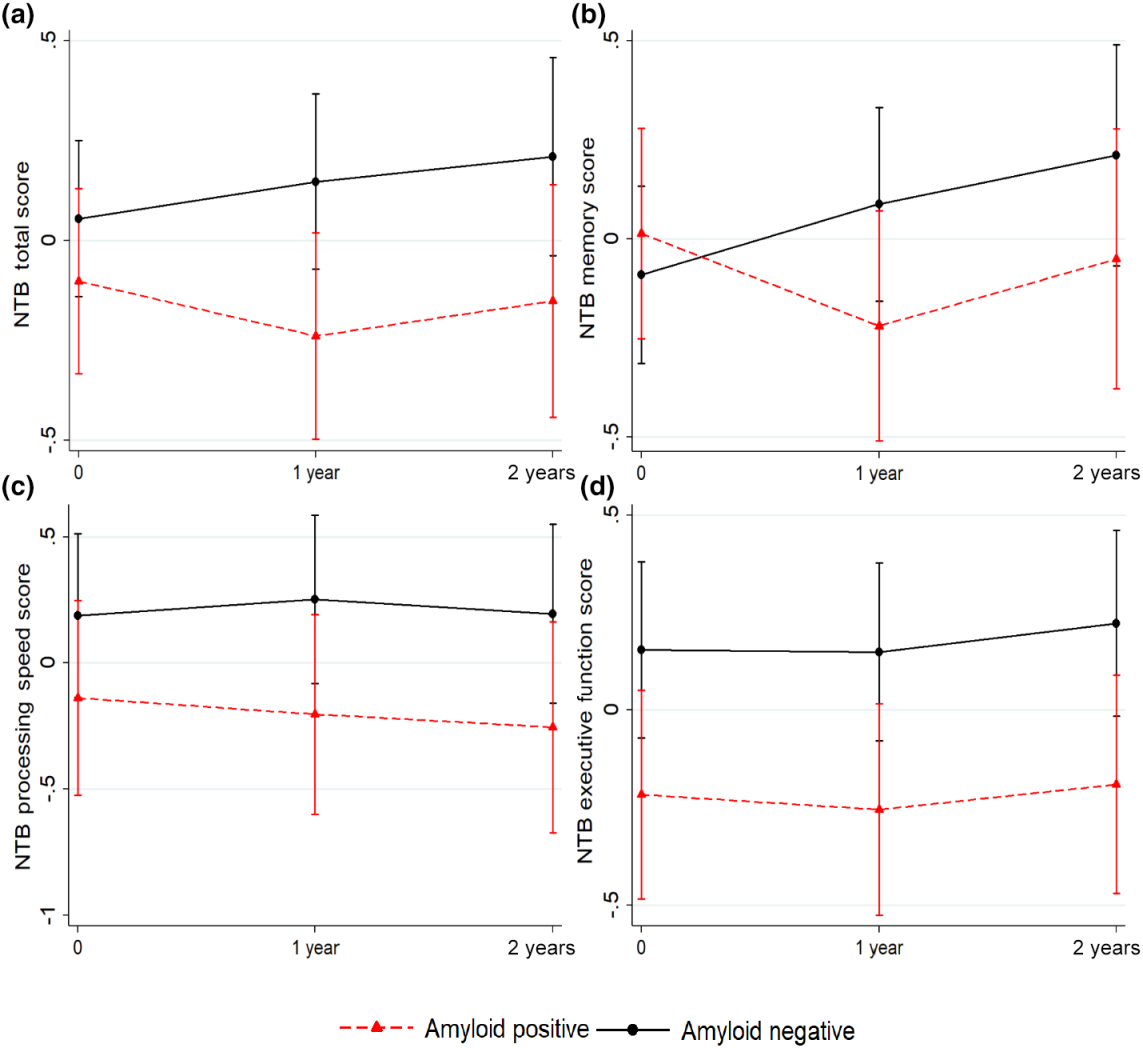
Baseline amyloid status and change in objective cognitive performance. A graphical representation shows change in cognitive performance over time (a, Neuropsychological Test Battery [NTB] total score; b, NTB memory score; c, NTB processing speed score; d, NTB executive function score) for the amyloid‐positive and amyloid‐negative groups. The figure shows estimated means of cognitive scores at baseline, 1 year, and 2 years, with higher scores indicating better cognitive performance. Error bars are confidence intervals. The differences between amyloid groups regarding cognitive change from baseline to 2 years were assessed with the mixed effects regression models.

Participant characteristics are shown in Table S1. Baseline Aβ+ status was significantly associated with lower baseline executive function in the main model (Table 1) and after adjustment for sex. Estimates were slightly lower after adjustment for age or education, and they were no longer statistically significant (Table 1). No other associations were found between Aβ**±** status and baseline NTB measures. Baseline Aβ+ status was significantly related to poorer performance over time for NTB total and memory scores in the main model (Table 1, Figure 1). For change in NTB memory, this association remained significant after further adjustment for age, sex, or education. For change in NTB total score, estimates were similar after further adjustment, although the 95% CI became slightly broader and included the null value in the age‐adjusted model (Table 1). No significant associations were found between baseline Aβ**±** status and baseline or longitudinal change in SCC (results not shown).

**TABLE 1:** Associations between baseline amyloid status and objective cognitive performance.

Objective cognitive measures	Main model	Main model with further adjustments		
		Age	Education	Sex
NTB total score				
Baseline	−0.20 (−0.51, 0.11)	−0.11 (−0.39, 0.16)	−0.11 (−0.39, 0.17)	−0.18 (−0.49, 0.13)
Change over time	**−0.10 (−0.20, −0.01)**	−0.08 (−0.17, 0.01)	**−0.10 (−0.20, −0.001)**	**−0.12 (−0.21, −0.02)**
NTB memory score				
Baseline	−0.03 (−0.32, 0.37)	0.12 (−0.20, 0.44)	0.12 (−0.20, 0.45)	0.08 (−0.26, 0.42)
Change over time	**−0.18 (−0.34, −0.03)**	**−0.15 (−0.30, −0.01)**	**−0.19 (−0.35, −0.04)**	**−0.20 (−0.35, −0.05)**
NTB processing speed score				
Baseline	−0.35 (−0.85, 0.16)	−0.24 (−0.73, 0.24)	−0.28 (−0.79, 0.22)	−0.33 (−0.84, 0.18)
Change over time	−0.06 (−0.23, 0.10)	−0.04 (−0.21, 0.12)	−0.04 (−0.21, 0.12)	−0.08 (−0.24, 0.09)
NTB executive function score				
Baseline	**−0.38 (−0.70, −0.05)**	−0.30 (−0.62, 0.01)	−0.28 (−0.58, 0.02)	**−0.39 (−0.72, −0.07)**
Change over time	−0.02 (−0.13, 0.09)	0.00 (−0.11, 0.11)	−0.01 (−0.12, 0.11)	−0.02 (−0.14, 0.09)

*Note*: For estimates and 95% confidence intervals in bold, *p*‐value < 0.05. Estimates and 95% confidence intervals are shown from mixed effects regression models with maximum likelihood estimation. In the main model, change in cognitive scores was analyzed as a function of baseline amyloid status (±), amyloid status × time interaction, randomization group, time, and group × time interaction. The amyloid status term shows the association between amyloid status and cognition at baseline, whereas the amyloid status × time interaction term shows the association between baseline amyloid status and change in cognition over time. Further adjustments for age, education, or sex also included their impact on change in cognition over time (age, age × time; education, education × time; sex, sex × time interactions).

Abbreviation: NTB, Neuropsychological Test Battery.

## DISCUSSION

This study examined the links between brain amyloid and objective and subjective cognitive measures in a trial‐ready at‐risk general population. Aβ accumulation showed some associations with objective but not subjective cognition. There was little difference in baseline cognition between Aβ± groups. However, the 2‐year trajectories of memory and global cognition were less favorable in the Aβ+ group. Interestingly, the impact of Aβ+ status on objective memory change did not translate into a similar impact on SCC, although SCC have been suggested as one of the first AD symptoms [4].

Although 42% of individuals in this general at‐risk population were Aβ+ at baseline, there was little cognitive decline during 2 years. This is in line with previous reports that many asymptomatic Aβ+ people can remain cognitively stable over time [2]. According to IWG 2021, AD diagnosis is clinical–biological, requiring both clinical symptoms and positive AD biomarkers. Our results do not indicate a clear link between Aβ accumulation and SCC; that is, SCC alone may not be a reliable early AD symptom. SCC plus other features not assessed in this study (e.g., duration, worry, informant confirmation) have been proposed as more indicative for preclinical AD [4].

Because this study included a trial‐ready general at‐risk population, our findings are especially important for AD/dementia prevention trials testing early interventions in Aβ+ individuals without substantial cognitive impairment. Baseline cognitive performance alone was not very different between Aβ+ and Aβ− groups. Although Aβ+ status seemed to affect cognitive trajectories, a 2‐year timespan, now common in many clinical trials, may not be sufficient to detect significant cognitive decline.

The FINGER trial recruited individuals with various risk factors for dementia but without substantial cognitive impairment, providing the opportunity to study Aβ accumulation at a very early at‐risk stage. The main limitation of the present study is the small sample size, restricting statistical power. Participants in the PET substudy were similar to the FINGER population but slightly older, which may have influenced results, because SCC are more common with ageing. Data on tau pathology markers were not available. Moreover, visual rating (common in clinical settings) was used to determine Aβ status, as there is currently no commonly accepted cutoff for Aβ pathology on PET scans. The 2‐year period was too short for the at‐risk participants to develop dementia, and ongoing extended follow‐up will determine dementia status.

In conclusion, brain Aβ accumulation may affect cognition from a very early at‐risk stage, but substantial cognitive decline will likely require a longer time to occur. Further studies with larger populations and longer follow‐up time are needed to accurately predict the rate of decline at the individual level.

## AUTHOR CONTRIBUTIONS

**Gazi Saadmaan:** Writing – original draft; formal analysis; writing – review and editing. **Anette Hall:** Formal analysis; writing – review and editing. **Tiia Ngandu:** Conceptualization; data curation; investigation; writing – review and editing. **Nina Kemppainen:** Data curation; investigation; writing – review and editing. **Francesca Mangialasche:** Writing – review and editing. **Gayle M. Wittenberg:** Writing – review and editing. **Anna Matton:** Writing – review and editing. **Juha O. Rinne:** Conceptualization; resources; writing – review and editing. **Miia Kivipelto:** Conceptualization; funding acquisition; writing – review and editing; supervision. **Alina Solomon:** Conceptualization; funding acquisition; supervision; writing – review and editing.

## FUNDING INFORMATION

This work was supported by the European Research Council (grant 804371); a research collaboration between Finnish Institute for Health and Welfare, Karolinska Institute, and Janssen Pharmaceutica (research project “Prediction modelling, identification and validation of novel molecular signatures of dementia”); EU Joint Program–Neurodegenerative Disease Research EURO‐FINGERS and Multi‐MeMo grants; an ERA PerMed Pattern‐Cog grant; a NordForsk NJ‐FINGERS grant; Alzheimerfonden (Sweden); the Swedish Research Council; Region Stockholm ALF (Sweden); the Center for Innovative Medicine at the Karolinska Institute (Sweden); Stiftelsen Stockholms Sjukhem (Sweden); the Knut and Alice Wallenberg Foundation (Sweden); the Swedish Research Council for Health, Working Life, and Welfare (FORTE); the Research Council of Finland; the Juho Vainio Foundation (Finland); Kela (Finland); the Ministry of Education and Culture (Finland); the Sigrid Jusélius Foundation (Finland); and the Alzheimer's Research and Prevention Foundation (USA). Janssen Research & Development provided input in study design and data interpretation. Other funding sources had no role in the design and conduct of the study; in the collection, analysis, and interpretation of the data; or in the preparation and review of the manuscript.

## CONFLICT OF INTEREST STATEMENT

G.M.W. is an employee of Janssen Research & Development. The other authors report no disclosures.

## Supporting information


Data S1.

## Data Availability

Data used in this study are not publicly available for ethical and legal reasons, but the data are available upon request. Those fulfilling the requirements for viewing confidential data as required by Finnish legislation and the Finnish Institute for Health and Welfare are able to access the data after completion of a material transfer agreement. Requests may be directed to kirjaamo@thl.fi.
