# Supplementary material for: Brain amyloid load, subjective memory complaints, and cognitive trajectories in older individuals at risk for dementia
Source: Eur J Neurol. 2024 Aug 12;31(12):e16436. doi: 10.1111/ene.16436 (PMC11554848; doi:10.1111/ene.16436)
Supplement: Supplementary file 1 — Data S1. [file ENE-31-e16436-s001.docx]

**Supplementary text 1.** **FINGER PiB-PET sub-study methods**

As previously published in Kemppainen et al., Neurology. 2018 Jan 16;90(3):e206-e213. doi: 10.1212/WNL.0000000000004827.

Participants in the FINGER exploratory neuroimaging sub-study were selected from the most recently recruited trial participants at the time when MRI/PET resources became available and if there were no contraindications. PiB-PET was available only at the Turku PET Centre in southwestern Finland.

In total, 48 individuals from the Turku cohort of the FINGER main study population were included in the PET sub-study. All 48 participants met the inclusion criteria for the FINGER main study^1^. Specifically, they had an increased risk for dementia based on the CAIDE Dementia Risk Score and demonstrated cognitive performance level that was at or slightly below the mean expected for their age, as assessed by the Consortium to Establish a Registry for Alzheimer’s Disease (CERAD) test. Participants were required to meet at least one of the following criteria: word list memory task results of ≤19 words, word list recall of ≤75%, or Mini-Mental State Examination (MMSE) score of ≤26 points. Mean MMSE score was 27 range 22–30, SD 1.8). Participants who had been diagnosed with dementia or those suspected of having dementia following a clinical assessment by a study physician at the FINGER screening visit (suggested further investigations) were excluded.

The FINGER PET sub-study population was similar to the rest of the Turku cohort and the overall FINGER main study population in terms of education years, proportion of APOE Ɛ4 carriers, and other characteristics (e.g., cardiovascular and metabolic risk factors). However, the FINGER-PET population was slightly older than the rest of the FINGER population. This age difference is attributable to a slight delay in the recruitment process for the Turku cohort; the birth years did not differ between the study populations.

In addition to visual assessment, a composite PiB retention score was calculated as the mean of the prefrontal, parietal, precuneus, anterior cingulate, posterior cingulate, and lateral temporal regions^5^.

Supplementary Table 1: Baseline sociodemographic and clinical characteristics of the participants by amyloid status on PiB-PET scans

| **Baseline population characteristics** | **Amyloid negative**  **n=28** | **Amyloid positive**  **n=20** | **P-value** |
| --- | --- | --- | --- |
| Age, years | 70.2 (5.9) | 71.6 (3.6) | 0.34 |
| Women, n (%) | 14 (50%) | 8 (40%) | 0.49 |
| Education, years | 9.7 (2.9) | 8.9 (2.1) | 0.32 |
| Baseline PiB composite score | 1.3 (0.07) | 1.8 (0.39) | 0.00 |
| **Objective cognitive measures** | | | |
| NTB total score | 0.04 (0.54) | -0.09 (0.51) | 0.41 |
| NTB memory score | -0.11 (0.53) | 0.03 (0.65) | 0.40 |
| NTB processing speed score | 0.16 (0.97) | -0.10 (0.79) | 0.32 |
| NTB executive function score | 0.16 (0.60) | -0.22 (0.45) | 0.02 |
| **Subjective memory complaints**† | | | |
| Prospective memory | 16.5 (3.69) | 17.8 (3.87) | 0.28 |
| Retrospective memory | 17.2 (4.47) | 17.9 (4.23) | 0.59 |
| Total memory | 33.7 (7.80) | 35.9 (7.95) | 0.38 |

Numbers are means (SDs) unless otherwise specified. P values are shown from t-test for continuous variables and Pearson ꭓ^2^ test for categorical variables.

For objective cognitive measures, higher values indicate better performance. For subjective memory complaints, higher values indicate more complaints.

†Number of participants with available data 47 for prospective memory, 44 for retrospective memory, 44 for total memory

NTB= Neuropsychological test battery

**References:**

1. Kivipelto M, Solomon A, Ahtiluoto S, et al. The Finnish Geriatric Intervention Study

to Prevent Cognitive Impairment And Disability (FINGER): study design and

progress. Alzheimers Dement 2013;9:657–665. DOI: 10.1016/j.jalz.2012.09.012

2. Harrison J, Minassian SL, Jenkins L, Black RS, Koller M, Grundman M. A neuro-

psychological test battery for use in Alzheimer disease clinical trials. Arch Neurol

2007;64:1323–1329. DOI: 10.1001/archneur.64.9.1323

3. Ngandu T, Lehtisalo J, Levalahti E, et al. Recruitment and baseline characteristics of

participants in the Finnish Geriatric Intervention Study to Prevent Cognitive Im-

pairment and Disability (FINGER): a randomized controlled lifestyle trial. Int J

Environ Res Public Health 2014;11:9345–9360. DOI: 10.3390/ijerph110909345

4. Matthews DR, Hosker JP, Rudenski AS, Naylor BA, Treacher DF, Turner RC. Homeostasis model assessment: insulin resistance and beta-cell function from fasting

plasma glucose and insulin concentrations in man. Diabetologia 1985;28:412–419. DOI: 10.1007/BF00280883

5. Kemppainen N, Johansson J, Teuho J, et al. Brain amyloid load and its associations with cognition and vascular risk factors in FINGER Study. *Neurology*. 2018;90(3):e206-e213. DOI:10.1212/WNL.0000000000004827
